# Predicting the influence of homologous recombination repair deficiency genes on glioma heterogeneity and patient prognosis using multi-omics analysis and machine learning

**DOI:** 10.1371/journal.pone.0337731

**Published:** 2025-12-19

**Authors:** Xin Wu, Longyuan Li, Zheng Zhan, Mei Chang, Jiaxuan Li, Zhouqing Chen, Zhong Wang

**Affiliations:** Department of Neurosurgery and Brain and Nerve Research Laboratory, The First Affiliated Hospital of Soochow University, Suzhou, China; The Second Affiliated Hospital, Chongqing Medical University, CHINA

## Abstract

**Background:**

Glioma is the most common malignant tumor of the central nervous system, and homologous recombination deficiency (HRD) may play a crucial role in its progression. Our study aimed to predict the impact of HRD on glioma heterogeneity and patient prognosis from a multi-omics perspective.

**Methods:**

We integrated HRD-related gene expression levels and survival information from The Cancer Genome Atlas (TCGA) and Chinese Glioma Genome Atlas (CGGA) databases. Using a combination of machine learning algorithms, we identified the optimal algorithm and constructed the HRD Index model. After validating the model’s accuracy, we assessed the expression heterogeneity of HRD-related genes in vitro using quantitative polymerase chain reaction (qPCR). Multiple omics analyses, including enrichment analysis, genomics, prediction of immune cell subtype infiltration, and drug sensitivity, were employed to demonstrate the heterogeneity and clinical predictive significance of the HRD Index in glioma.

**Results:**

Through algorithm selection, the LASSO-RSF (Least Absolute Shrinkage and Selection Operator – Random Survival Forest) algorithm identified 7 genes (POLR2F, FANCB, PTEN, PLK3, INO80D, PRMT6, and UNG) to construct the HRD Index. Model validation demonstrated excellent accuracy. qPCR results revealed differential expression of these HRD Index genes among different cell lines. Samples grouped by HRD Index showed potential differences in certain cytokine and receptor pathways, as well as varying gene mutation frequencies between groups. Drug sensitivity analysis indicated that the HRD Index could predict treatment efficacy for specific drugs.

**Conclusion:**

Our HRD Index model based on these seven genes significantly correlated with clinical prognosis in glioma patients and holds promise for guiding clinical management.

## Introduction

Gliomas are the most common tumors originating in central nervous system [1]. In 2021, the World Health Organization (WHO) published the fifth version of the Classification of Tumors of the Central Nervous System, the adult-type diffuse gliomas were divided into glioblastoma (IDH-wildtype), oligodendroglioma (IDH-mutant, and 1p/19q-codeleted), and astrocytoma (IDH-mutant) [2]. Unlike the fourth version, the classification principle of the fifth version paid more attention to the molecular genetics [3]. Among the several factors that lead to mutations, DNA damage and repair play an extremely important role [4]. Several direct or indirect factors such as ionizing radiation or certain chemotherapy drugs, can cause various types of DNA damage includes single-strand breaks, double-strand breaks (DSB), or other types [5]. DNA damage usually impairs DNA stability, and loss of this stability is an important primary cause in the development of many types of cancer [6].

Normally, cells have evolved a series of complex response mechanisms to combat factors that threaten genomic integrity and even use DNA damage to create new opportunities for natural selection, which are known as DNA damage response (DDR) [7]. DDR can be divided into a series of distinct but functionally linked pathways, which are mainly determined by the type of DNA damage [8,9]. In the process of DNA repair, the repair of DSBs is crucial for maintaining genomic stability [10]. Two pathways play a crucial part in the repair of DSB: non-homologous end joining (NHEJ) and homologous recombination repair (HRR) [11]. HRR is an evolutionarily conserved repair modality [12], in this process, a homologous gene is selected as a template, and its sequence is copied to the damaged region for repair, when the template is near the damaged region, this mechanism allows the gene sequence to be accurately repaired, restoring the DNA to the genetic form before DSB occurs [13]. Defects in the pathway of HRR was known as homologous recombination repair defects (HRD), it can greatly affect the maintenance of gene integrity, especially when large-scale gene damage occurs [14].

Loss of heterozygosity (LOH), large-scale transitions (LST) and telomeric allelic imbalance (TAI) have been identified as features of genomic instability in which HRR is defective [15]. Severe genetic damage can initiate apoptosis—a programmed cell death pathway [16]. When tumors are treated with drugs that target DNA damage, this defective mechanism may render these tumors more sensitive to these therapeutic regimens [17]. This phenomenon has been demonstrated in ovarian cancer, where previous clinical studies have shown thatovarian cancer patients with HRD have a favorable response rate to poly (adenosine diphosphate-ribose) polymerase inhibitor (PARPi), particularly among patients who have a clinical response to platinum-based chemotherapy, the addition of PARPi may provide a substantial progression-free survival benefit [18].

In the treatment of glioma, temozolomide (TMZ), as a first-line chemotherapeutic agent, also targets genetic damage to kill tumor cells [19]. However, the efficient DNA repair in response to TMZ is frequently linked to drug resistance, especially in high-grade glioma patients [20]. The mechanism of this resistance emerges may be related to gene repair processes present in glioma cells since that knockdown or blockage of DNA repair gene promoters has been found to reduce DNA repair capacity and increase TMZ-mediated DNA damage [21]. Thus, understanding the reasons of therapy resistance and discovering medications that improve the therapeutic efficacy of current therapies may help glioma patients survive longer.

Recent advances in machine learning and artificial intelligence have seen broad uptake in glioma research [22–24]. To probe the contribution of HRD to treatment response and prognosis, we compiled 149 HRD-related genes from published studies and computed an HRD index to stratify risk in patients with glioma.

## Method

### Data collection

All data we collected in this study were from public datasets. The RNA-seq data and corresponding clinical follow-up of glioma patients were obtained from a combined cohort of The Cancer Genome Atlas (TCGA, https://portal.gdc.cancer.gov/). We extracted the data of glioma patients in the Chinese Glioma Genome Atlas (CGGA, http//www.cgga.org.cn/) as external validation. For differential analysis, we used the COUNTS normalized matrix from the aforementioned database, while for other bioinformatics analyses, we utilized the FPKM normalized matrix. The entire data is normalized to ensure error-free access to the next step.

### Cell culture

Glioma cell lines U87 and U251 were obtained from the Shanghai Institute of Biochemistry and Cell Biology, and authentication was performed using short tandem repeat (STR) DNA analysis. The cells were cultured in Dulbecco’s Modified Eagle’s Medium (DMEM) supplemented with 10% fetal bovine serum (FBS, obtained from Shanghai Zhongqiao Xinzhou Biotechnology Co., Ltd.) and maintained in a 37°C incubator. Cell morphology was observed daily, and the culture medium was refreshed regularly.

### Collection of patient-derived glioma samples

We included glioblastoma patients who underwent surgical operations at The First Affiliated Hospital of Soochow University from May 2025 to September 2025. Tumor core samples resected during surgery and peritumoral samples removed during extended resection were preserved. Pathological examination was performed on the obtained samples, and patients with pathology reports confirming glioblastoma were included in the study. All patients were informed preoperatively that their tumor samples might be used in research and provided written informed consent. The samples obtained during surgery were immediately transferred to a −80°C freezer for preservation and then moved to liquid nitrogen storage the following day. The samples included in this study were obtained from The First Affiliated Hospital of Soochow University and were approved by the Ethics Committee of The First Affiliated Hospital of Soochow University, with the ethics approval number: 2025−247.

For this study, we retrospectively included tumor core and peritumoral samples from four patients. Total RNA was extracted from these samples using NcmZol Reagent (New Cell & Molecular Biotech Co., Ltd, M5100), and Real-Time PCR was performed as described previously.

### HRD-related genes collection and construction of a prognostic model

We collected 133 previously published HRD-related genes (S1 Table) and obtained their expression levels in the TCGA and CGGA cohorts. We utilized Random Forest, Gradient Boosting Machine, Lasso regression, Enet, StepCox, Survival SVM, SuperPC, and CoxBoost algorithms for machine learning analysis. In addition to applying these eight algorithms individually, we also constructed combination models by pairing the algorithms. In each combination, the important features identified by the first algorithm were used to build the model with the second algorithm, resulting in a total of 70 combinations. Based on the C-index ranking, we selected the optimal algorithm to establish an HRD-related gene prognostic model (HRD Index) in the TCGA and CGGA cohorts. Subsequently, to more visually assess the risk level of each patient, we calculated the HRD Index for each patient based on the coefficients and expressions, and divided the patients into high and low risk groups according to maximally selected rank satistics (A statistical method used to select the optimal cutpoint, typically applied for the segmentation of continuous variables). The HRD Index for each patient is calculated as follows:


$$\begin{array}{c} HRD\;Index\;=\;\sum {\mathrm{\backslash nolimits}}_{k=\text{1}}^{n}coef({mRNA}^{K})*\text{exp}({mRNA}^{k}) \end{array}$$

The abbreviation coef in the formula is the regression coefficient of the screened mRNA associated with survival, while the abbreviation exp is its expression.

### Validation of prognostic models

After grouping, the model was combined with patient survival time and survival status through survival curves to determine the grouping ability of HRD Index. Scatter plots and box plots were also used to evaluate grouping. Time-dependent ROC curves were plotted based on patient survival data to assess the predictive effectiveness of the model. Patient survival time is counted by days and the outcome event for survival status is death. To validate the applicability of the model in the in-house dataset, we calculated the HRD Index for each sample in TCGA dataset as well as differentiated the risk stratification of the patients. Fair internal stratification ability of the model as evaluated by survival curves, scatter plots and box plots. To further prevent overfitting and to assess the validity of the model in the external real world, CGGA data was introduced as an external validation set and the same operation was performed as above.

### Introduction of clinical attributes and calibration of nomogram

We substituted individual clinical data including age, gender, IDH mutation, MGMT methylation, 1p19q coding, and pathology into the model and performed subgroup analysis to construct a forest plot. Meanwhile, the ROC curves of each data were derived. The t test was used to generate correction curves to test whether the predicted results matched the actual. The nomogram which predicts overall patient survival at 1, 3, and 5 years was created. Decision curve analysis (DCA) was used to assess the fit of the model.

### Immune infiltration and immune checkpoint analysis

To explore the relationship between predictive models and the tumor immune microenvironment, we compared immune checkpoint activation between high and low scoring groups and analyzed immune and stromal scores using the ESTIMATE R package. The immune infiltration statuses between the different groups were calculated using CIBERSORT (http://cibersort.stanford.edu/), a tool based on the principle of linear support vector regression that deconvolutes expression matrices of human immune cell subtypes.

### Differential gene analysis and enrichment analysis

Differential gene expression analysis was conducted using the “DESeq2” package on sequencing counts data from glioma samples in the TCGA and CGGA cohorts across different groups. Genes with a fold change greater than 2 and an adjusted p-value (FDR) less than 0.05 were selected as significantly upregulated or downregulated. These genes were then subjected to GO and KEGG enrichment analyses. The top five pathways from each enrichment analysis based on counts were included for visualization.

### Real-time reverse transcription PCR (qRT-PCR)

For glioma cell lines, lysis was performed using PIPA strong lysis buffer (Beyotime, China, P0013B). After collecting the lysate, total mRNA was extracted using an mRNA extraction kit (Sangon Biotech, M5102) and stored at −20°C for short-term storage. Reverse transcription was carried out using ABScript Neo RT Master Mix for qPCR with gDNA Remover (RK20433). The qPCR analysis was performed using Hieff® qPCR SYBR Green Master Mix (No Rox) (11201ES03) following the manufacturer’s instructions. S2 Table provides the primer sequences for constructing the HRD Index genes. The results were standardized using the 2^(-ΔΔCt) method and visualized with GraphPad Prism 10.

### Transfection

The Si-FANCB used in this study was sourced from RiboBio (Guangzhou, China). The experiment was conducted in 6-well plates, with each well plated with approximately 0.5 ml of Opti-MEM medium containing 25 μl/104 U87 cells. A total of 30 μl of Lipofectamine 2000 (Thermo Fisher Scientific, Waltham, MA, USA) was diluted in 1 ml of Opti-MEM medium. Then, 20 μl of Si-FANCB was mixed with 40 μl of the diluted Lipofectamine 2000 and 0.5 ml of Opti-MEM medium to prepare the working solution, which was then added to the 6-well plates. The plates were incubated in the cell culture incubator for 48 hours, after which RT-PCR was performed to assess transfection efficiency.

### CCK-8 assay

After 48 hours of transfection, the cells from the 6-well plates were collected and transferred to a 96-well plate, with approximately 10^6 cells per well. The plate was placed in a 37°C incubator for further incubation. At 1, 2, 3, and 4-day intervals, 10 μl of CCK-8 solution (Beyotime, China) was added to each well of the 96-well plate, and the plate was then incubated for an additional 2 hours. The absorbance at 450 nm for each well was measured using a microplate reader.

### Transwell assay

Migration and invasion assays for U87 cell lines were performed using Transwell chambers with 8 μm pore polycarbonate membranes and 24-well plates. The Transwell chambers were inserted into the 24-well plates, and cell suspensions containing 4 × 104 cells in serum-free medium were added to the chambers. The wells of the 24-well plates were filled with DMEM containing 20% FBS. After incubating for 24 hours at 37°C, cells remaining in the upper chamber were removed, and the cells that adhered to the outer membrane were fixed with methanol and stained with crystal violet. The stained cells were gently rinsed with tap water. Cell migration was assessed and imaged using a microscope at 200X magnification. For the invasion assay, the procedure was similar to the migration assay, except that the Transwell chambers were coated with Matrigel.

### DEPMAP

CRISPR (Depmap Public 24Q2+score, chronos) and Expression Public 24Q2 data for glioma cell lines were obtained from the DEPMAP database (https://depmap.org/portal/). This database provides comprehensive information on gene expression, mutations, copy number variations, and methylation across 1,379 cell lines. Additionally, it includes data on the importance of target genes in various cell lines screened using CRISPR-Cas9 technology, where a lower score indicates higher gene importance.

### Tumor mutation burden and copy number analysis

We used the “TCGAbiolinks” package to download the “Masked Copy Number Segment” and “Masked Somatic Mutation” for glioma patients in the TCGA cohort and utilized the maftools package to annotate and visualize DNA mutations in the samples. Copy number variations were annotated using the gistic2.0 function on the GenePattern website and visualized in R.

### Drug sensitivity analysis

We used the “pharmacoGx” package to download drug sensitivity data from the Cancer Cell Line Encyclopedia (CCLE, pSet name: CCLE_2015). Subsequently, we used the “OncoPredict” package to predict tumor drug sensitivity. By using the half-maximal inhibitory concentration (IC50) of drugs in glioma from the CCLE and RNA sequencing data as the training set, we predicted the IC50 for each sample in the TCGA and CGGA cohorts using their RNA sequencing data. The Spearman method was employed to analyze the correlation between the HRD Index and the IC50 of different drugs.

## Results

### Establishment of HRD index using machine learning algorithms

**Fig 1:**
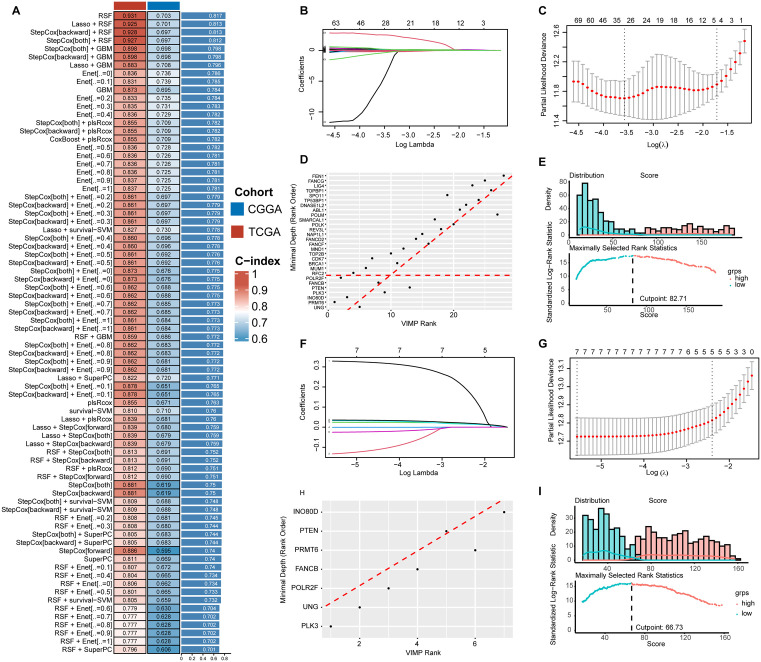
Constructing an HRD-related gene prognostic model. A: Selection of the optimal algorithm combination from 77 machine learning algorithms. B: Log(lambda) sequence plot of HRD-related genes obtained through LASSO regression in the TCGA cohort. C: Distribution of LASSO coefficients for HRD-related genes in the TCGA cohort. D: Identification of the most important genes in the RSF algorithm using the VIMP method and minimal depth method in the TCGA cohort. E: Classification of TCGA samples into high HRD Index and low HRD Index groups based on predictive scores. F: Log(lambda) sequence plot of HRD-related genes obtained through LASSO regression in the CGGA cohort. G: Distribution of LASSO coefficients for HRD-related genes in the CGGA cohort. H: Identification of the most important genes in the RSF algorithm using the VIMP method and minimal depth method in the CGGA cohort. I: Classification of CGGA samples into high HRD Index and low HRD Index groups based on predictive scores. HRD: Homologous recombination repair defects; TCGA: The Cancer Genome Atlas; CGGA: Chinese Glioma Genome Atlas.

After employing various combinations of machine learning algorithms, LASSO combined with Random Survival Forest (RSF) exhibited the highest C-index in both the TCGA and CGGA cohorts (Fig 1A). Initially, LASSO regression was used to screen HRD-related genes in the TCGA cohort. Genes with prognostic value were then further selected using RSF (Fig 1B, C), ultimately identifying seven genes: POLR2F, FANCB, PTEN, PLK3, INO80D, PRMT6, and UNG (Fig 1D). We determined the HRD index for each sample using the maximum ranking selection statistic (Fig 1E, S3 Table). Validation of these seven genes in the CGGA cohort also yielded positive results, and the HRD index for each sample in the CGGA cohort was established (Fig 1F–I, S4 Table). Accuracy testing of the HRD Index in the TCGA and CGGA cohorts at 1-year, 2-year, and 3-year time points showed consistently high accuracy (S1A–D Fig). Additionally, stratification of TCGA and CGGA cohort samples based on the median expression levels of these seven genes revealed significant differences in survival Kaplan-Meier curves between high and low expression groups (S2 Fig).

### Validation of HRD Index predictive accuracy through clinical information and phenotype

**Fig 2:**
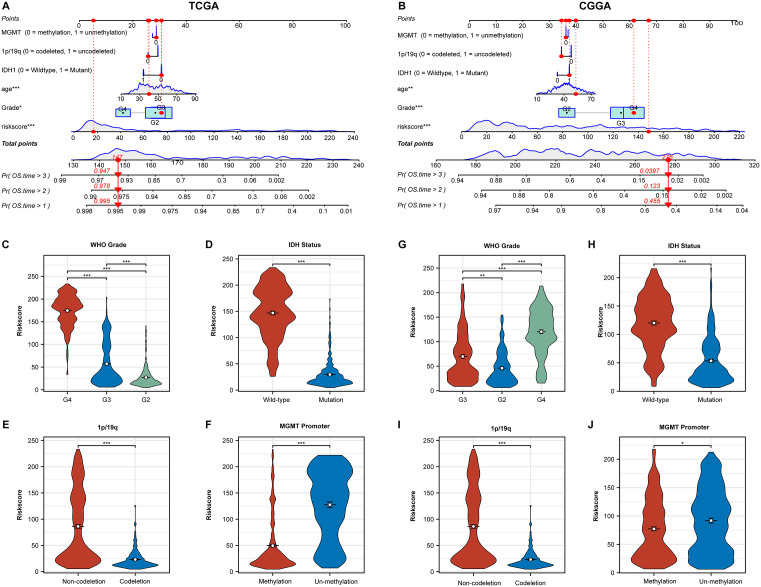
Construction of a nomogram incorporating clinical information. A: Nomogram constructed using the TCGA cohort, incorporating MGMT promoter methylation status, 1p/19q co-deletion status, IDH1 mutation status, age, and WHO grade. B: Nomogram constructed using the CGGA cohort, incorporating MGMT promoter methylation status, 1p/19q co-deletion status, IDH1 mutation status, age, and WHO grade. C-F: Box plots depicting differences in HRD Index among groups stratified by WHO Grade **(C)**, IDH1 mutation status **(D)**, 1p/19q co-deletion status **(E)**, and MGMT promoter methylation status (F) in the TCGA cohort. G-J: Box plots depicting differences in HRD Index among groups stratified by WHO Grade **(G)**, IDH1 mutation status **(H)**, 1p/19q co-deletion status **(I)**, and MGMT promoter methylation status (J) in the CGGA cohort. * p < 0.05; ** p < 0.01; *** p < 0.001.

We collected clinical information and phenotypic data for each sample in the TCGA and CGGA cohorts, including IDH mutation status, MGMT promoter methylation status, 1p/19q co-deletion status, age, and WHO grade. We constructed a nomogram incorporating these factors along with the HRD Index (Fig 1A, B). The HRD Index demonstrated significant accuracy in predicting prognosis. Furthermore, we visualized the differences in HRD Index across different clinical subgroups. It was observed that gliomas marked as WHO grade 4 had a significantly higher HRD Index compared to the other two groups, and samples marked as WHO grade 3 had a higher HRD Index than those marked as grade 1. Additionally, in subgroups associated with poorer prognosis (IDH-wildtype vs. IDH mutation, 1p/19q non-codeletion vs. 1p/19q codeletion, MGMT promoter unmethylated vs. MGMT promoter methylated), the HRD Index was significantly elevated (Fig 2C–F). These results were also validated in the CGGA cohort (Fig 2G–J).

### Differences in HRD index across various glioma cell lines

**Fig 3:**
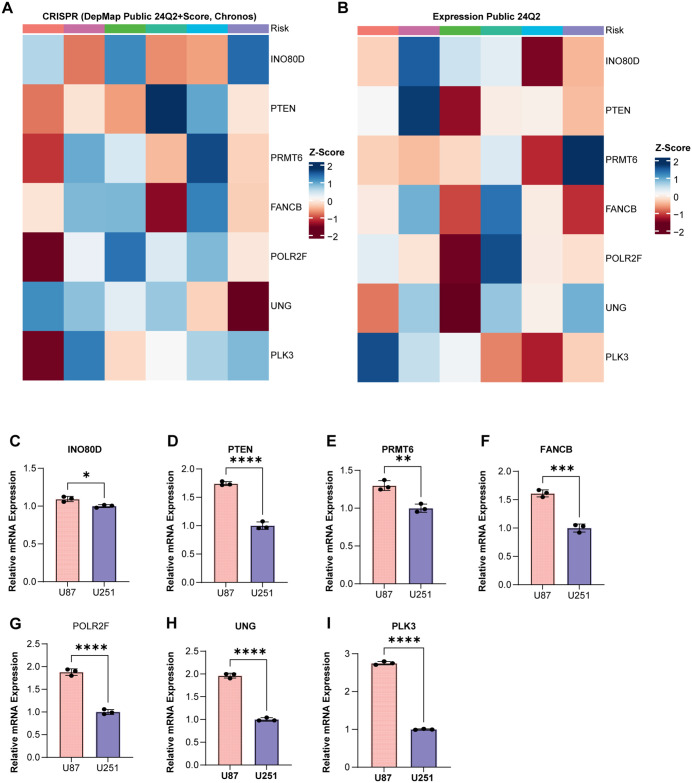
Differences in HRD index across various cell lines. A: Importance of genes involved in constructing the HRD Index in different cell lines (Z-score normalization, with red indicating high importance and blue indicating low importance). B: Expression levels of genes involved in constructing the HRD Index in different cell lines (Z-score normalization, with red indicating low expression and blue indicating high expression). C-I: qPCR analysis of the expression levels of genes involved in constructing the HRD Index in U87 and U251 cell lines. * p < 0.05; ** p < 0.01; *** p < 0.001.

**Fig 4:**
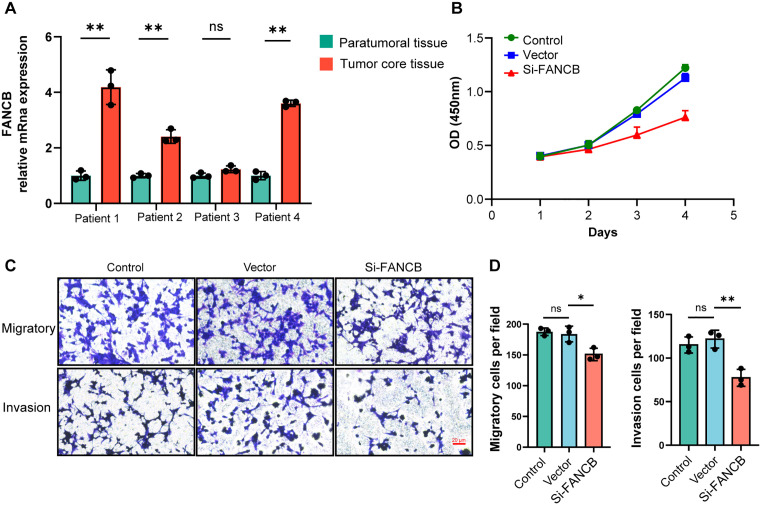
Intervening with FANCB in gliomas can inhibit the proliferation and migration capabilities of glioma cells. A: Expression levels of FANCB in patient-derived tumor core samples and adjacent non-tumor samples, measured by RT-PCR across different groups. B: Proliferation capacity of U87 cells in the Control, Vector, and si-FANCB groups. C: Migration and invasion abilities of U87 cells in the Control, Vector, and si-FANCB groups. D: The number of migrating and invading cells per field in U87 cell lines. * p < 0.05; **p < 0.01; ***p < 0.001.

We first analyzed seven genes involved in constructing the HRD Index using the DEPMAP database. From the CRISPR (Depmap Public 24Q2+score, chronos) data, it is evident that the importance of HRD-related genes varies across different cell lines (Fig 3A). Additionally, the expression levels of genes contributing to the HRD Index differ among cell lines, suggesting the presence of distinct HRD Index profiles in different cell lines (Fig 3B). These findings were further validated through in vitro experiments. The qPCR results also showed a similar trend (Fig 3C–I), with notably lower expression levels of HRD Index genes in the U87 cell line. These results highlight the heterogeneity of the HRD Index across glioma samples and suggest that this heterogeneity could be a significant factor contributing to survival differences among patients. To further investigate the role of this phenomenon in human glioma, we collected paired specimens from the tumor core and peritumoral regions of four patients. Quantitative PCR showed that FANCB expression was higher in the tumor core than in the peritumoral tissue (Fig 4A). In vitro, downregulation of FANCB reduced tumor cell proliferation (Fig 4B) and attenuated invasive and migratory capacities (Fig 4C–D).

### Enrichment analysis between different HRD index groups

**Fig 5:**
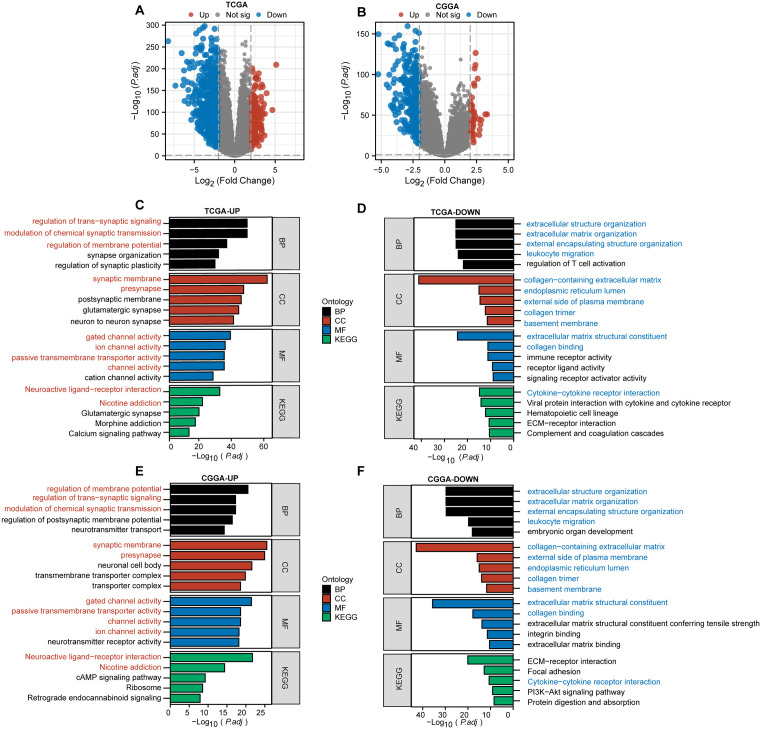
Differential analysis and pathway enrichment analysis between different HRD Index groups. A-B: Volcano plots of differentially expressed genes between HRD Index groups in the TCGA cohort (A) and the CGGA cohort **(B)**. C: GO and KEGG enrichment analysis of upregulated genes in the TCGA cohort. D: GO and KEGG enrichment analysis of downregulated genes in the TCGA cohort. E: GO and KEGG enrichment analysis of upregulated genes in the CGGA cohort. F: GO and KEGG enrichment analysis of downregulated genes in the CGGA cohort. Pathways enriched in both the TCGA and CGGA cohorts for upregulated genes are highlighted in red. Pathways enriched in both cohorts for downregulated genes are highlighted in blue. GO: Gene Ontology; CC: Cellular Component; BP: Biological Process; MF: Molecular Function; KEGG: Kyoto Encyclopedia of Genes and Genomes.

We performed differential analysis on samples categorized into high HRD Index and low HRD Index groups within the TCGA and CGGA cohorts. The results showed a total of 1,557 upregulated genes and 2,365 downregulated genes in the TCGA cohort (Fig 5A, S5 Table). In the CGGA cohort, there were 870 upregulated genes and 1,317 downregulated genes (Fig 5B, S6 Table). We conducted GO and KEGG enrichment analyses on these upregulated and downregulated genes (Fig 5C–F).

The results indicated that several pathways were enriched in both the TCGA and CGGA cohorts. Upregulated genes were often enriched in receptor-related pathways, such as neuroactive ligand-receptor interaction and gated channel activity (Fig 5C, E). Conversely, downregulated genes were predominantly enriched in cytokine-related pathways, such as cytokine-cytokine receptor interaction (Fig 5D, F). This suggests that differences in HRD-related genes may be associated with certain ion channel activities and intercellular communication.

### Differences in gene mutations between different HRD index groups

**Fig 6:**
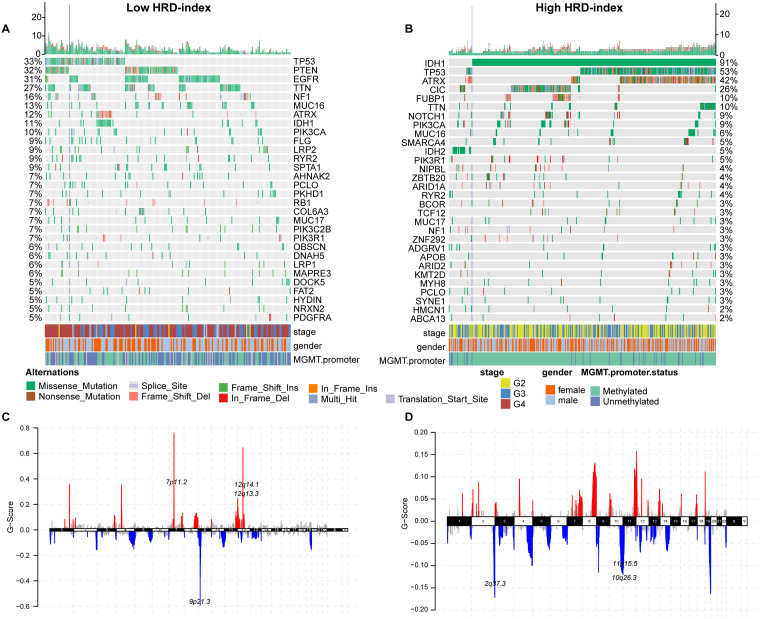
Genomic differences between different HRD index groups. A: Top 30 mutated genes in the high HRD Index group. B:Top 30 mutated genes in the low HRD Index group. C: Copy number variations in the high HRD Index group. D: Copy number variations in the low HRD Index group.

To observe genomic differences between the different HRD Index groups, we visualized the top 30 genes with the highest mutation frequencies in the high HRD Index and low HRD Index samples (Fig 6A, 6B). In the high HRD Index group, the gene with the highest mutation frequency was TP53 (approximately 33%), whereas in the low HRD Index group, the highest mutation frequency was observed in IDH1 (approximately 91%). According to the 2021 WHO classification of central nervous system tumors, IDH1 mutations are indicative of lower-grade gliomas, which might explain why samples with higher HRD Index are associated with poorer prognosis (Fig 2A).

Additionally, the high HRD Index group showed higher mutation frequencies in PTEN (approximately 32%), EGFR (approximately 31%), TTN (approximately 27%), and NF1 (approximately 16%) (Fig 6A, 6B). This increased mutation frequency may be related to the higher probability of gene mutations following HRD. Chromosomal analysis also revealed different mutation sites in the high HRD Index group (Fig 6C, 6D).

### Differences in immune cell infiltration and immune checkpoint expression between different HRD index groups

**Fig 7:**
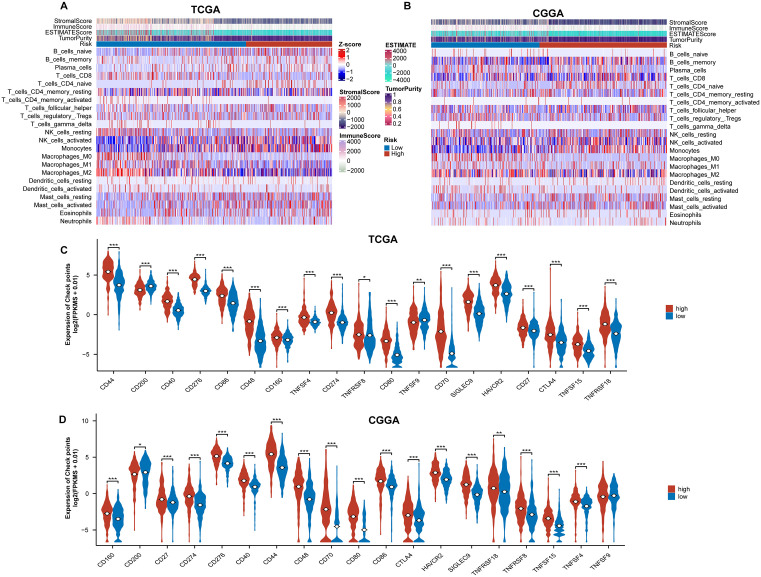
HRD Index predicts immune cell infiltration. A-B: Immune infiltration analysis between different HRD Index groups in the TCGA cohort (A) and CGGA cohort **(B)**. C-D: Expression levels of negative immune checkpoints’ mRNA between different HRD Index groups in the TCGA cohort (C) and CGGA cohort **(D)**. *p < 0.05; **p < 0.01; ***p < 0.001.

By predicting the extent of immune cell infiltration in samples from the TCGA and CGGA cohorts, we created immune cell infiltration profiles for the different groups (Fig 7A, B). Samples marked with a higher HRD Index exhibited higher tumor purity and lower Stromal, Immune, and ESTIMATE scores. Additionally, the high HRD Index group showed lower infiltration of CD8 + T cells and higher infiltration of NK cells. These variations in immune cell infiltration could be one of the reasons for the differing prognosis between the two groups (Fig 7A, B).

We also analyzed the expression levels of immune checkpoints between different groups. The results showed significant differences in the mRNA expression levels of genes marked as negative immune checkpoints in both the TCGA and CGGA cohorts (Fig 7C, D). These findings suggest that HRD may influence tumor immune regulation processes.

### HRD index and treatment response to targeted therapies

**Fig 8:**
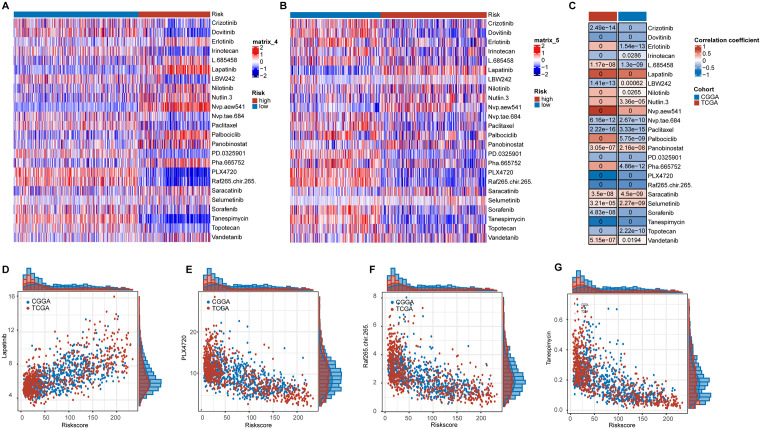
HRD Index predicts targeted therapy efficacy. A-B: Heatmap of predicted drug sensitivity for targeted therapies in the TCGA cohort (A) and CGGA cohort **(B)**. C: Heatmap showing the correlation between HRD Index and sensitivity to various drugs in the TCGA and CGGA cohorts. D-G: Correlation between drug sensitivity and HRD Index for the four drugs with a correlation greater than 0.5 in both the TCGA and CGGA cohorts.

We predicted the treatment responses of different samples to various targeted therapies in the TCGA and CGGA cohorts. The results showed significant differences in treatment responses for almost all drugs between the two groups in both TCGA and CGGA cohorts (Fig 8A, B). Subsequently, we conducted correlation analyses between drug sensitivity and HRD Index across different drugs, revealing consistent correlation trends in both TCGA and CGGA cohorts (Fig 8C). Specifically, Lapatinib, PLX4720, Raf265.chir.265, and Tanespimycin exhibited significant correlations (r > 0.5) with HRD Index in both cohorts (Fig 8D–G). These findings suggest that HRD Index may serve as a predictive biomarker for treatment responses to these drugs.

## Discussion

In order to analyze the role if HRD in the clinical prognosis of glioma patients in detail, we developed a novel predictive model. In this study, we collected 133 genes from previous literature and their expression in TCGA glioblastoma (n = 174) and low-grade glioma (n = 532) cohorts. We intersected Lasso-Cox regression model and random forest model to obtain a prediction robust model based on seven HRD-related genes (UNG, PRMT6, INO80D, PLK3, PTEN, FANCB, POLR2F). To investigate the prognostic value of these seven genes in glioma, we calculated the HRD index for each patient based on the coefficients and expressions, and combined the model with different clinical characteristics of patients.

Generally speaking, DDR can be divided into a series of distinct but functionally linked pathways, which are mainly determined by the type of DNA damage. Two pathways play important roles in the repair of double-stranded DNA breaks: NHEJ and HRR [11,17]. After the occurrence of DSB, the broken DNA ends were cleaved into two single-strands, the 3’ end of which is paired with the homologous template to form a D-loop structure, in this process, RAD51 binds to the broken DNA ends and mediates the binding of homologous templates [7], subsequently RAD51 breaks from the leading strand of DNA and exposes its 3 ‘end, and this sequence is used as a primer for DNA synthesis [25]. This type of repair tends to restore the DNA to its original state [26]. NHEJ occurs throughout the cell cycle. Previous studies have pointed out that HRR and NHEJ have competing mechanisms for repairing DSB, and NHEJ plays a more important role in DNA repair when HRD is present [27]. This will inevitably lead to the occurrence of genomic instability events [13]. Some scholars have pointed out that mutations in genes involved in the HR pathway may lead to susceptibility to glioma [28]. But the effects of this defect in gene repair are not all negative. Recent clinical study showed that PARP inhibitors of bevacizumab treatment of advanced ovarian cancer group has obvious progression-free survival benefit, especially in HRD positive tumor patients [18], in addition, in the treatment of ovarian cancer, as a kind of predictive biomarkers, HRD has used to identify can benefit from bevacizumab and olaparib combination therapy of patients with ovarian cancer [29].

Therefore, HRD can be regarded as a defect that can potentially affect the prognosis of cancer, and the effect may be double-faced, on the one hand, it can guide the drug use of cancer patients, and on the other hand, it may lead to an increase in the probability of mutation. In our selection of seven HRD-related genes, the high expression of PLK3, PRMT6, UNG, and FANCB is associated with poorer prognosis in glioma patients, whereas the high expression of POLR2F, INO80D, and PTEN is linked to better prognosis. In different cell lines, CRISPR-mediated knockout of these genes yields varied impacts on cell survival. For instance, genes like UNG and PLK3 play a significant role in most glioma cell lines, whereas PTEN and INO80D exhibit a weaker influence in the majority of cell lines. Previous literature suggests that UNG and PTEN are crucial in the occurrence of HRD [30,31]. However, in the glioma patients from the TCGA and CGGA cohorts, the expression of these two genes shows an inverse relationship with prognosis. This phenomenon suggests that although the presence of HRD in tumors may enhance the therapeutic efficacy of certain treatments, such as PARP inhibitors [32], the roles of individual HRD-related genes in tumors are not identical. According to the nomogram, the HRD-index we constructed serves as an independent prognostic factor, indicating that a comprehensive scoring system may better reflect the overall effect of HRD in gliomas.

After constructing the HRD-index to evaluate the impact of HRD-related genes on glioma prognosis, we explored the differences among various HRD-index groups. In terms of mutation frequency, the high HRD-index group exhibited more mutations in genes like PTEN and EGFR, whereas IDH1 mutations predominantly occurred in the low HRD-index group. This suggests that different levels of HRD may correlate with key genetic mutations in gliomas.

Moreover, the significance of the HRD-index in glioma extends beyond prognosis prediction. Immune infiltration analysis revealed distinct differences in immune cell infiltration levels across HRD-index groups, with higher infiltration of CD8 + T cells and M1-polarized macrophages, both of which are involved in antitumor responses, in the low HRD-index group [33,34]. Additionally, certain negative immune checkpoints were significantly upregulated in samples with a low HRD-index. These findings suggest that the HRD-index might also serve as a predictive marker for immune cell infiltration or immune cell activity within the glioma tumor microenvironment.

In terms of drug sensitivity prediction, the relationship between different drugs and the HRD-index varied. For instance, the high HRD-index group showed higher IC50 values for EGFR-targeting drugs such as Erlotinib, Lapatinib, and Vandetanib [35–37], indicating poorer therapeutic outcomes. This could be related to the higher frequency of EGFR mutations in the high HRD-index group. Conversely, drugs that interfere with DNA replication or cell division, such as Irinotecan and Topotecan, had lower IC50 values in the high HRD-index group [38,39], suggesting potentially better therapeutic outcomes. These results align with the underlying mechanism of HRD, further validating the accuracy of our HRD-index and indicating its potential for predicting therapeutic responses to various treatments.

The main limitation of this study is the lack of positive identification of certain well-established genes, such as BRCA1/2, RAD51C, and PALB2, which play crucial roles in HRD [40–42]. This may be due to the inherent complexity of studying tumors at the molecular level. In addition, because our analyses were performed at the mRNA level, the results may not capture the prognostic effects of these genes at the protein level. Despite these issues, we believe the model remains informative: HRD-related genes engage in complex interactions, and our framework provides an mRNA-level interpretation of HRD phenomena in glioma. In our approach, we used computational methods to correlate HRD-related pathway genes with the clinical features of gliomas and validated the results using external datasets and clinical genome sequencing from glioma samples of different grades. While this approach can uncover potential relationships that are often overlooked, it cannot fully explain the roles these genes play in glioma cells, which remains a key limitation of our study. With respect to immune infiltration, we did not provide direct histologic evidence from patient-derived tissue sections to substantiate our predictions; rather, we analyzed database-sourced samples using in silico inference. The results in this area should therefore be interpreted with appropriate caution given potential methodological bias. Additionally, despite careful analysis during model construction, the study included only 1,223 glioma cases, which may not comprehensively represent the general glioma population. Future research with larger datasets will be needed to better generalize the findings. Although there are challenges, we remain dedicated to further research in this field. Understanding the complexities of tumor genetics is a profound and ongoing challenge for all researchers. While it may take years or even lifetimes to uncover these mysteries, it is a pursuit that continues to drive scientific progress and inspire hope for breakthroughs.

## Supporting information

S1 Fig Accuracy testing of the HRD Index in the TCGA and CGGA cohorts at 1-year, 2-year, and 3-year time points. (TIF)

S2 Fig Survival Kaplan-Meier curves between high and low expression groups. (TIF)

S1 Table The list of HRD-related genes included in this study. (XLSX)

S2 Table The primer sequences for constructing the HRD Index genes. (DOCX)

S3 Table The HRD index for each sample in the TCGA cohort. (XLSX)

S4 Table The HRD index for each sample in the CGGA cohort. (XLSX)

S5 Table Up-regulated genes and down-regulated genes in the TCGA cohort. (XLSX)

S6 Table Up-regulated genes and down-regulated genes in the CGGA cohort. (XLSX)
