# Supplementary figures and images for: Predicting the influence of homologous recombination repair deficiency genes on glioma heterogeneity and patient prognosis using multi-omics analysis and machine learning
Source: PLoS One. 2025 Dec 19;20(12):e0337731. doi: 10.1371/journal.pone.0337731 (PMC12716779; doi:10.1371/journal.pone.0337731)

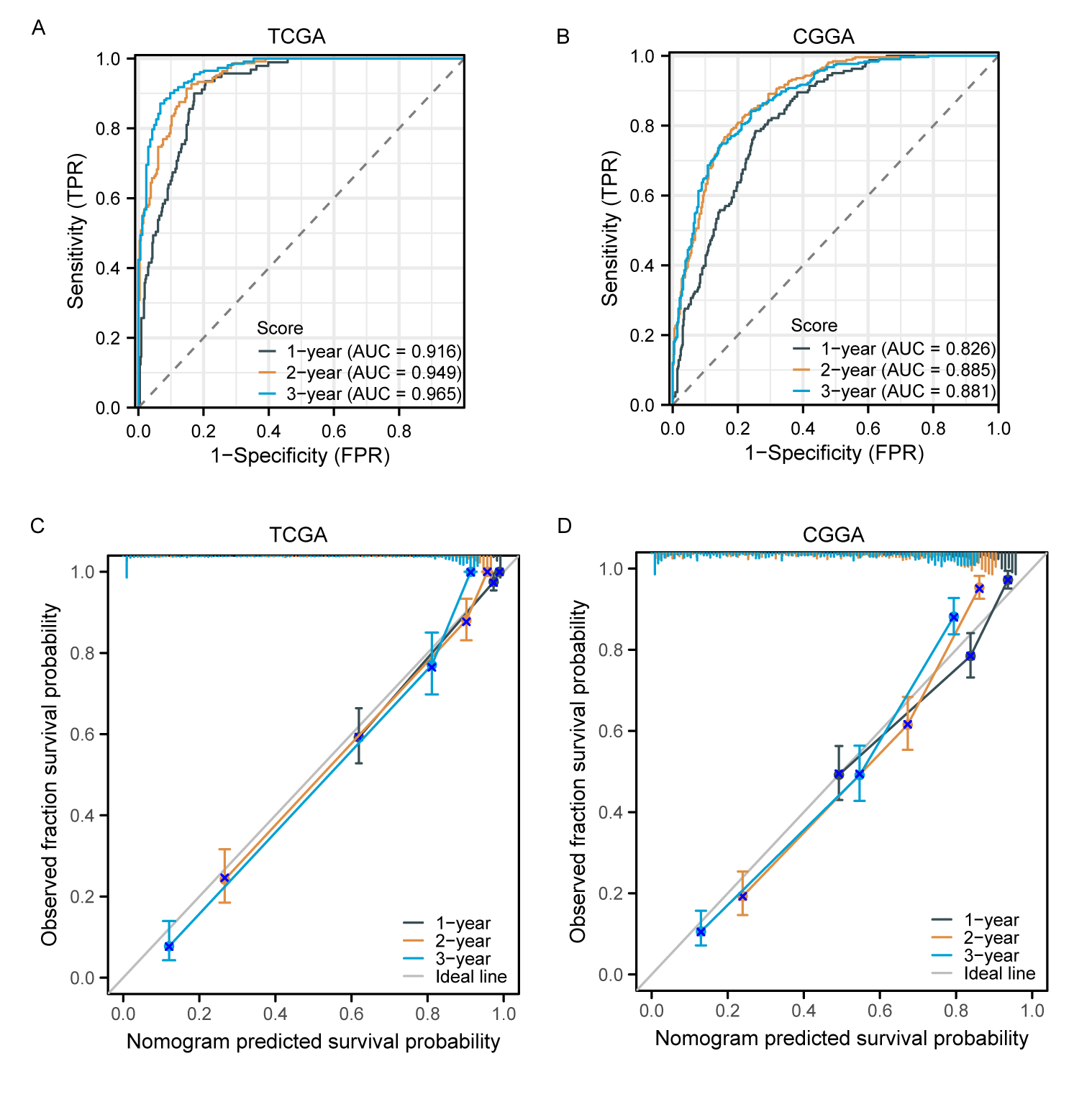

Supplement: S1 Fig — (TIF) [file pone.0337731.s001.tif]

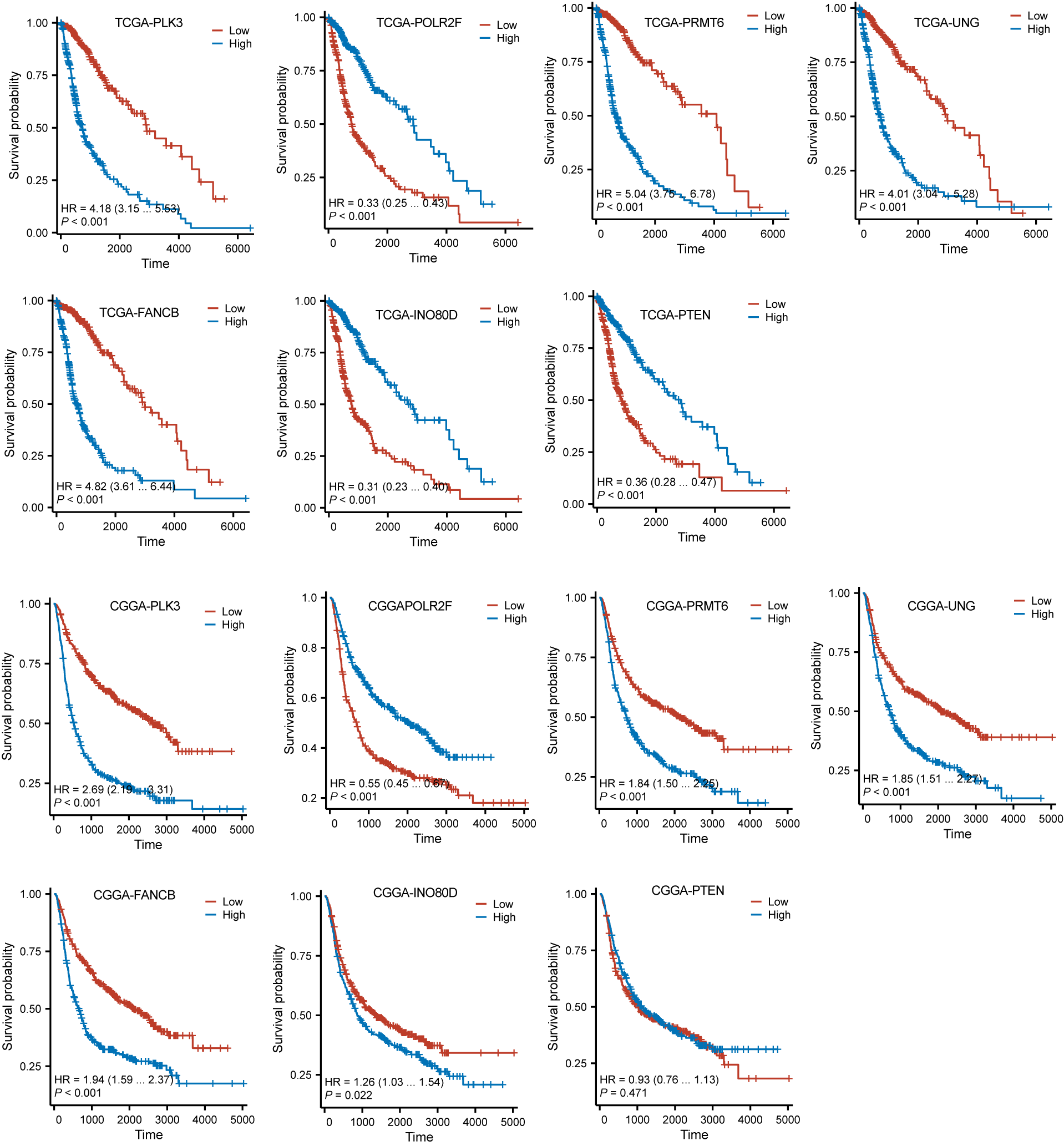

Supplement: S2 Fig — (TIF) [file pone.0337731.s002.tif]
