# Supplementary material for: Predicting the influence of homologous recombination repair deficiency genes on glioma heterogeneity and patient prognosis using multi-omics analysis and machine learning
Source: PLoS One. 2025 Dec 19;20(12):e0337731. doi: 10.1371/journal.pone.0337731 (PMC12716779; doi:10.1371/journal.pone.0337731)
Supplement: S2 Table — (DOCX) [file pone.0337731.s004.docx]

| PLK3 F | AGCGCCTACGCTGTCAAAG |
| --- | --- |
| PLK3 R | CTCAAAGTGGTGCGAAAAACG |
| PRMT6 F | TACCGCCTGGGTATCCTTCG |
| PRMT6 R | CCTGTTCCGGCAACTCTACA |
| POLR2F F | ATGTCAGACAACGAGGACAATTT |
| POLR2F R | TTCGGCATTCTCCAAGTCATC |
| UNG F | CCCCACACCAAGTCTTCACC |
| UNG R | TTGAACACTAAAGCAGAGCCC |
| INO80D F | ATAAGCCCTTGTGCTCATATAGC |
| INO80D R | AGCGTTGGCTGTTATACTTGG |
| FANCB F | ATGAAGGATGGCCTAAGGGTC |
| FANCB R | ACACACTAACAACTTTGCCAGT |
| PTEN F | ACACACTAACAACTTTGCCAGT |
| PTEN R | ACACACTAACAACTTTGCCAGT |
